# Field Longevity of a Fluorescent Protein Marker in an Engineered Strain of the Pink Bollworm, *Pectinophora gossypiella* (Saunders)

**DOI:** 10.1371/journal.pone.0038547

**Published:** 2012-06-05

**Authors:** Michelle Walters, Neil I. Morrison, John Claus, Guolei Tang, Caroline E. Phillips, Robin Young, Richard T. Zink, Luke Alphey

**Affiliations:** 1 Animal Plant Health and Inspection Service, Plant Protection and Quarantine, Centers for Plant Health Science and Technology, United States Department of Agriculture, Phoenix, Arizona, United States of America; 2 Oxitec Limited, Oxford, Oxfordshire, United Kingdom; 3 Strangeways Research Laboratory, Cardiovascular Epidemiology Unit, University of Cambridge, Cambridge, Cambridgeshire, United Kingdom; 4 Animal Plant Health and Inspection Service, Plant Protection and Quarantine, Centers for Plant Health Science and Technology, United States Department of Agriculture, Fort Collins, Colorado, United States of America; 5 Department of Zoology, University of Oxford, Oxford, Oxfordshire, United Kingdom; Natural Resources Canada, Canada

## Abstract

The cotton pest, pink bollworm (*Pectinophora gossypiella* (Saunders)), is a significant pest in most cotton-growing areas around the world. In southwestern USA and northern Mexico, pink bollworm is the target of the sterile insect technique (SIT), which relies on the mass-release of sterile pink bollworm adults to over-flood the wild population and thereby reduce it over time. Sterile moths reared for release are currently marked with a dye provided in their larval diet. There are concerns, however, that this marker fails from time to time, leading to sterile moths being misidentified in monitoring traps as wild moths. This can lead to expensive reactionary releases of sterile moths. We have developed a genetically marked strain that is engineered to express a fluorescent protein, DsRed2, which is easily screened under a specialised microscope. In order to test this marker under field conditions, we placed wild-type and genetically marked moths on traps and placed them in field cages. The moths were then screened, in a double-blind fashion, for DsRed2 fluorescence at regular intervals to determine marker reliability over time. The marker was shown to be robust in very high temperatures and generally proved reliable for a week or longer. More importantly, genotyping of moths on traps by PCR screening of the moths was 100% correct. Our findings indicate that this strain - and fluorescent protein markers in general - could make a valuable contribution to SIT.

## Introduction

The pink bollworm (*Pectinophora gossypiella* (Saunders)), originally native to Australia or Asia [1], [2], is a globally important pest of cotton. In south-western USA and northern Mexico, this moth has been the target of the Pink Bollworm Eradication Program, an area-wide, international effort to eliminate the pest from cotton (and its minor hosts). The sterile insect technique (SIT) [3] is a critical component of the PBW Eradication Program (http://www.cotton.org/tech/pest/bollworm/index.cfm). In pink bollworm SIT, the insect is mass-reared, marked internally by dye, sterilised with radiation and mass-released by air over cotton fields to find and mate with their wild counterparts. If sufficient steriles are released, the reduction in wild-to-wild mating results over time in population reduction [4], [5]. SIT has been particularly valuable in the San Joaquin Valley in California, where it prevented establishment of pink bollworm for over 40 years [6].

As the pink bollworm control programme progresses through its eradication phase, SIT continues to perform a significant role [7]. At this stage, the recapture rate of wild pink bollworm in monitoring traps (sticky Delta traps (Scentry Biologicals Inc.) baited with synthetic female sex pheromone) is very low [8] and accurate monitoring in the field is critical. Recapture of wild moths typically sparks significant and costly reactive sterile releases around the site of the ‘wild’ captures [9]. If a fraction of these wild captures are actually misidentified sterile moths, due to marker failure, such releases are a waste of resources. In addition, surviving progeny of released sterile moths would be indistinguishable from wild moths: lepidopteran SIT programmes generally irradiate with close to sub-sterilising doses to minimise radiation-related reduction in field performance. Although in moths the progeny of sub-sterilised adults will themselves be sterile – an effect known as F_1_ sterility [10] – they would be indistinguishable from fertile wild moths. Accurate marking of released moths and subsequent screening of those recaptured in traps is, therefore, increasingly important.

Current sterile moth marking relies on a red dye (Solvent Red 26, Royce International) added to the larval diet. This dye renders moth tissues a red colour [11], making them easily distinguishable from wild moths (which lack this colouring). Where the marking is weak, the moths can be homogenised and subjected to a chromatography test, which is more sensitive than visual screening. There remains, however, the possibility that the marker fails at a low rate if, for example, a moth excretes all the dye. SIT programme personnel have suggested that the longer a moth lives in the field, the less dye it carries [12]. When the dye marking is very weak, it can be difficult to detect in recaptured moths, even with a chromatography test.

**Figure 1:**
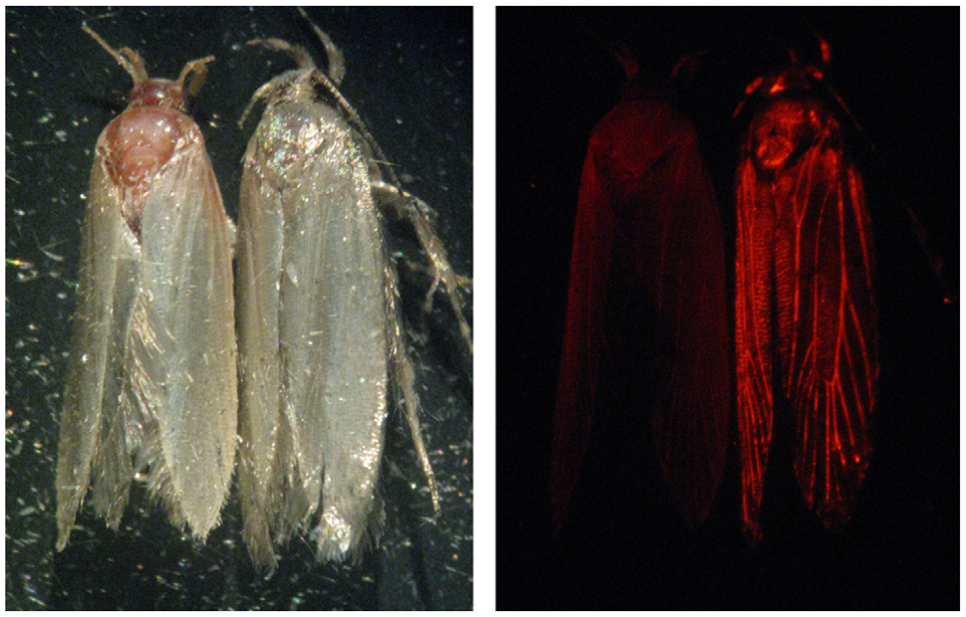
Photographs showing wild-type (left) and OX1138B (right) adult moths under bright field and DsRed2 excitation wavelength light, respectively.

To overcome this problem, an engineered strain of pink bollworm – called OX1138B [13] - was generated that expresses the DsRed2 fluorescent protein (Clontech Laboratories Inc.) [14], [15]. This marker can be detected by viewing under a suitable epi-fluorescence microscope (Figure 1), and can also be detected by PCR. In the first open-field trial of a genetically engineered insect, properties of OX1138B relevant to SIT effectiveness – pheromone response, dispersal and persistence of sterile males in cotton fields – were compared with the current wild-type SIT strain [13]. In all measures, the performance of both strains was similar, and in a demonstration SIT programme in south western Arizona the wild population was suppressed. The marker was easily screened and one potential failure of the dietary red marker was also detected.

**Figure 2:**
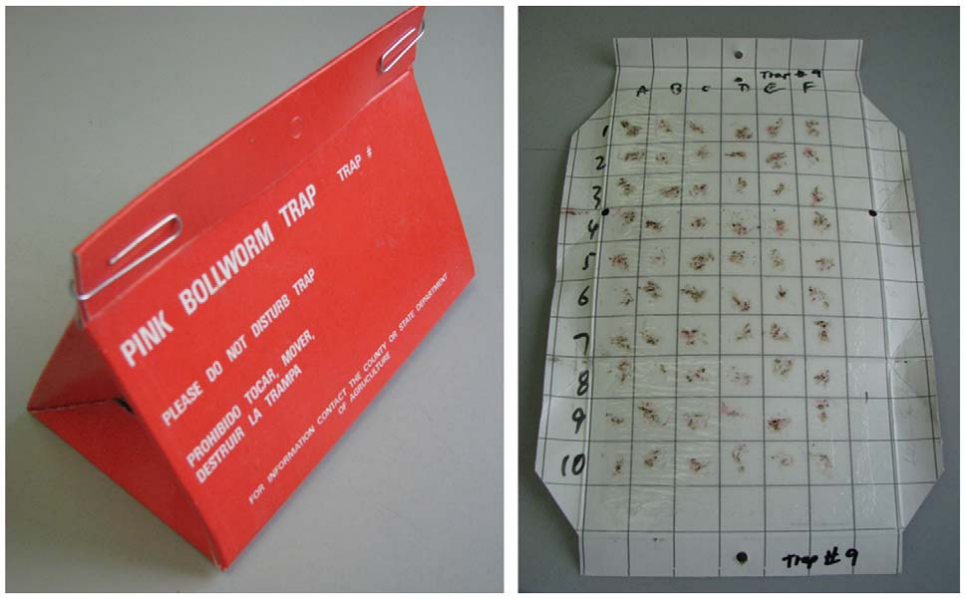
Delta trap: assembled (left) and opened (right) with grids marked A-F horizontally and 1-10 vertically, to hold 60 moths. The inner, white surface is coated with glue, which traps any insects that come into contact with it.

The performance of live moths in the field was the focus for these previous trials. While the data collected were encouraging, additional information on field performance of the DsRed2 marker in OX1138 is required before the strain can be considered for full programme use. We examined the robustness of the fluorescent protein marker in OX1138B, to assess its reliability on the sticky Delta traps used in the SIT programme and how this changed over time under field conditions. The experiment was conducted over four consecutive, approximately 1-month, periods in field cages, with all moths reared on Solvent Red, and a mixture of OX1138B and APHIS moths placed on Delta traps (Figure 2). Regular double-blind screening of the moths for the fluorescent marker was conducted over the course of each period, at the end of which each moth was PCR-genotyped.

## Results

**Figure 3:**
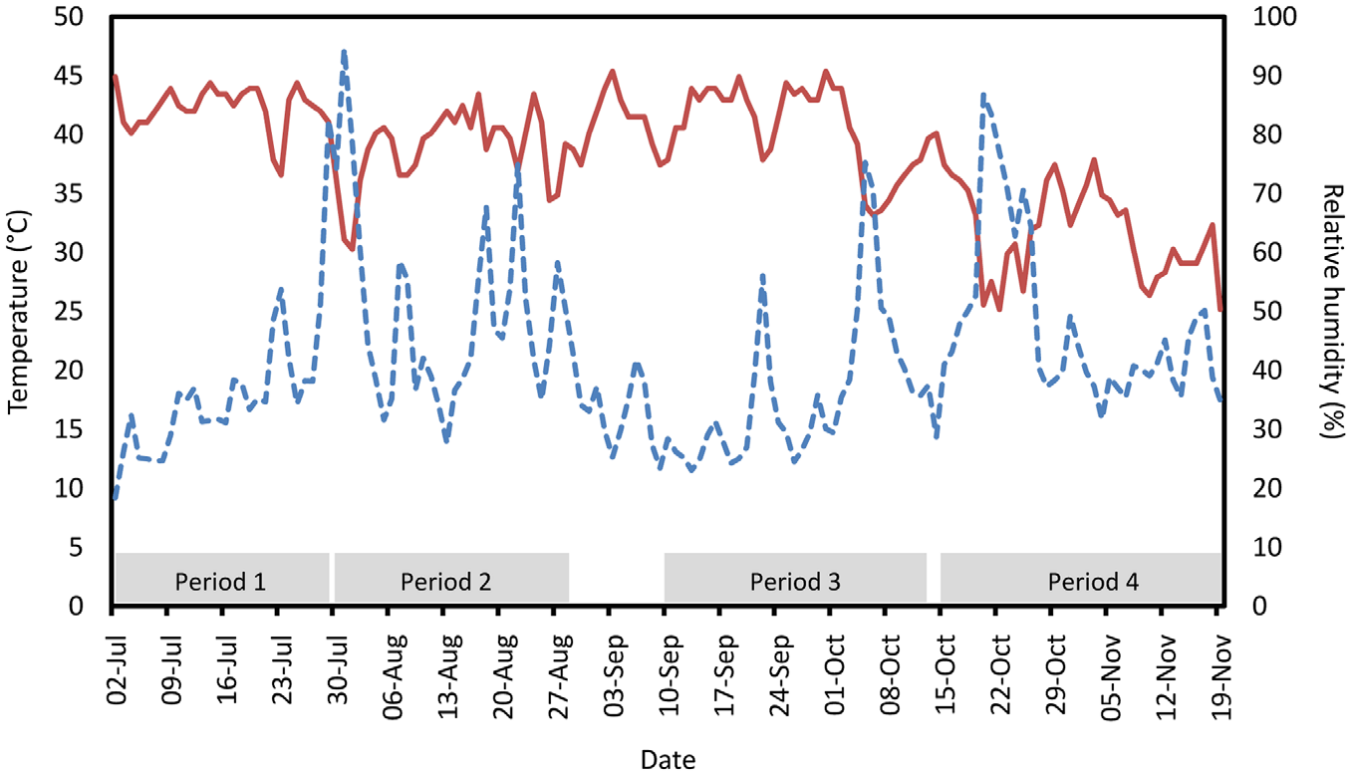
Maximum daily temperature (solid line) and mean daily relative humidity (dashed line) data recorded in a field cage over course of the experiment. The marked Periods indicate the duration of each experiment.

Over the course of the experiment, temperatures were recorded (Figure 3) in the cage. Temperatures remained high until Period 4, when it became generally cooler. Period 1 showed the highest temperatures (mean daily maximum, 42.3°C; standard deviation, 1.9°C), and these dropped slightly in Periods 2 and 3 (38.8°C±3.3°C and 40.5°C±3.6°C), and much more in Period 4 (31.6°C±3.8°C). Analysis of variance indicates daily maximum temperatures were significantly different between periods after correction for multiple testing (all p<0.001), apart from between Periods 1 and 3 (p = 0.193), and between Periods 2 and 3 (p = 0.078). Although the maximum daily temperatures in Period 1 were significantly higher than in Period 2, the mean values were high in both, and differed only by 3.5°C. With mean temperatures, this drop over time was more gradual: 34.5°C (±5.2°C) in Period 1, 31.8°C (±4.9°C) in Period 2, 28.5°C (±7.6°C) in Period 3 and 19.1°C (±7.9°C) in Period 4.

**Figure 4:**
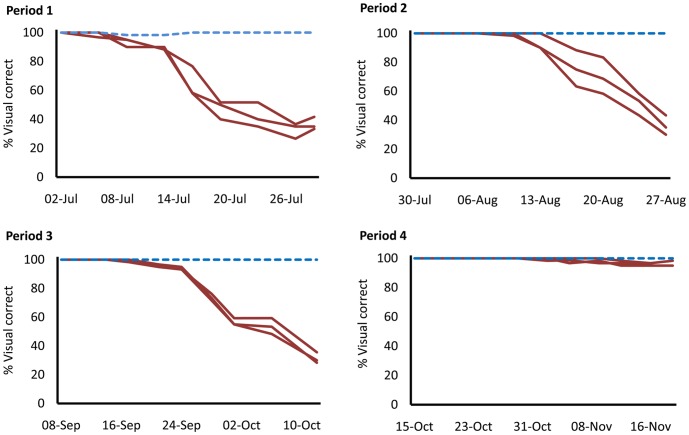
Reliability of DsRed2 fluorescent marker over time, during the four experimental periods. In each graph, the dashed line represents results from the trap kept in the laboratory at 26°C, and the solid lines represent traps kept in the field cage.

Relative humidity, another environmental factor that may influence marker longevity, was measured over the course of the four experimental periods (Figure 3). Mean daily relative humidity was 36.4% (±15.8%) in Period 1, 49.8% (±21.7%) in Period 2, 35.4% (±19.8%) in Period 3 and 47.9% (±23.8%) in Period 4. An analysis of variance comparison between Periods, after correction for multiple testing, indicates that mean relative humidity was significantly higher in Periods 2 and 4 than in Periods 1 and 3 (Periods 1 and 2, p = 0.001); Periods 1 and 4, p = 0.005; Periods 2 and 3, p<0.001; Periods 3 and 4, p = 0.001). In terms of DsRed2 screening, the traps retained in the laboratory at 26°C showed very little degradation in scoring accuracy in all periods (Figure 4). In fact, screening of these traps was 100% correct throughout, apart from early scoring during Period 1. These early screening failures and subsequent improvement suggest that it took a few days for the trap screener to become accustomed to screening for fluorescence.

**Figure 5:**
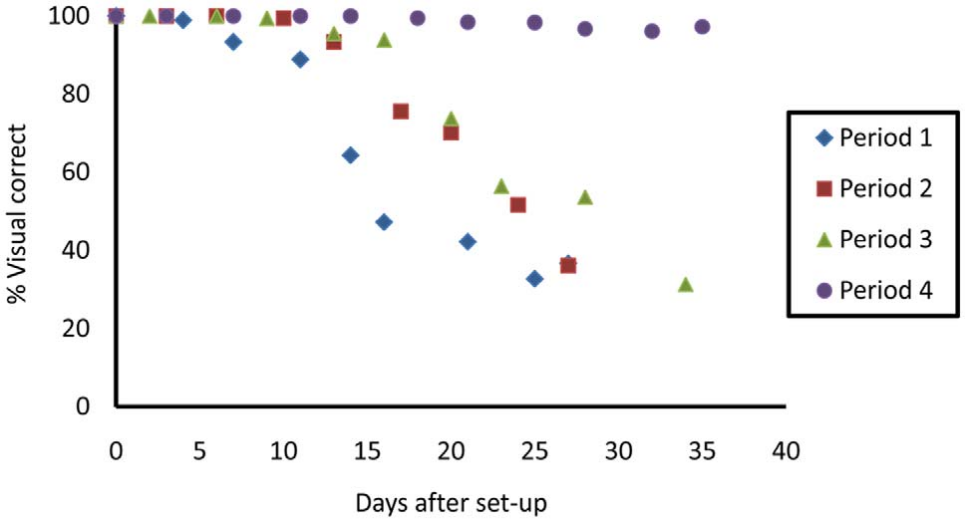
Mean reliability (traps combined) of DsRed2 fluorescent marker over time for Periods 1-4.

A logistic regression model adjusted for the days of each experiment suggests all Periods were significantly different from each other in terms of marker persistence (p<0.001 for all comparisons). Period 4 stands out as showing very little degradation throughout its duration (35 days) (Figure 5). Periods 2 and 3 show very similar marker persistence, with scoring reliability starting to decline from around 10 days. Period 1 showed more rapid decline in the marker compared to other periods, with the marker starting to fail in one trap at day 4 after set-up. The screening errors observed in traps kept at 26°C in Period 1, likely due to lack of prior experience on the part of the screener, indicate the potential for similar such error in the field cage traps during this period.

At the conclusion of the field cage experiment, moths from each period were genotyped by PCR, to identify them as either OX1138B or APHIS. Double-blind analysis of 253 OX1138B moths and 47 APHIS moths resulted in 100% correct PCR identification.

## Discussion

Our results show that the marker persists well under field conditions. Given weekly servicing of traps, which is the current eradication programme practice, DsRed2 would provide a highly reliable marker for SIT programmes. The fluorescent marker persisted much longer in laboratory conditions than in field cages. Several environmental differences could explain this, of which temperature and perhaps light intensity seem the most likely. In another setting, sunlight may also cause ‘bleaching’ of the protein’s fluorescent properties, but the transgenic moths in this experiment were sheltered from direct sunlight by the Delta trap and by some degree by the cage cover. Bacterial or fungal decomposition of the moths could have a significant impact on marker longevity, with humidity the most likely environmental factor to influence this microbial growth. Humidity was highest in Periods 2 and 4, when marker persistence was different, with reliability starting to drop after 10 days in the former and remaining very reliable (>96% correct) in the latter after 35 days in the field cage. The moths in the traps also showed little sign of decomposition throughout each period.

Therefore, temperature seems the most influential factor affecting persistence of the fluorescent marker. Moreover, the marked decline in daily temperatures in Period 4 corresponded with near-100% reliability of the DsRed2 marker for the duration of the period (35 days). The protein’s robustness in daily mean temperature peaks of 31.6°C, in field cage conditions, for 5 weeks greatly exceeds the required durability for an SIT programme: in the US, traps are typically collected after 1 week [16].

Marker persistence was seemingly lower in Period 1. However, the initial failures of the marker in the laboratory-stored trap and that trap’s subsequent reversion to 100% reliability, indicates an initial period of inexperience on the part of the trap screener. Results from Periods 2 and 3, when temperatures were still high, might be considered more reliable.

In Periods 2 and 3 the DsRed2 marker remained reliable (more than 99% correct identification) for 9–10 days in much hotter conditions than in Period 4. Again, this would provide the marker longevity required in an SIT programme.

Furthermore, the 100% reliability of PCR genotyping – the molecular marker – provides an extremely reliable, independent method, in case of uncertainty or for critical samples.

These results are encouraging for the prospects and value of integrating OX1138B, or other DsRed2-marked strains, into an SIT programme. The inclusion of OX1138’s reliable and heritable fluorescent marker, with the addition of its extremely robust molecular marker, would reduce or eliminate the need for expensive responses to false-positive captures of wild moths in monitoring traps. Genotyping by PCR can be undertaken in a few hours, so would provide rapid confirmation, even in samples that have been in the field for multi-week periods.

Other methods of marking have been considered, such as marking moths with radioactive isotopes [17], [18] or protein [19] applied in their larval diet, and genetic markers [20]–[22]. Methods where the marker is applied, for example isotopes in the feed, may suffer the same disadvantages as those of Solvent Red 26 dye – primarily uncertainty about reliability in a very large number of released insects – and may also reduce the performance of the moth in the field. Detection methods may also be expensive or time-consuming. Genetic markers might also be considered, for example the *sooty* mutation in pink bollworm [22], [23]. Although the *sooty* strain performed well in field and lab tests [20], it was unstable in mass-rearing: within months, only 70% of the *sooty* colony showed the mutant phenotype (E. Miller, personal communication). The various coloured-eye strains [24] also suffered the same problems in mass-rearing. These problems indicate that a transgenic marker may be the preferable approach for SIT.

For SIT, a gamma radiation dose of 200 Gy confers full sterility in the great majority of irradiated pink bollworm moths [25]. A very small proportion of these moths, however, produce offspring, of which the great majority are themselves fully sterile (F_1_ sterile). There remains, however, at least a theoretical possibility for releases of irradiated OX1138B to yield a very small number of fertile progeny. With respect to potential for the OX1138B strain to establish in the field, the existing measures to control wild pink bollworm – including SIT, *Bt* cotton and mating disruption – represent very significant obstacles. Furthermore, as the eradication programme proceeds, containment measures in the mass-rearing facility are being tightened for the APHIS strain. More generally, transgenesis imposes a fitness penalty [26]–[28]; this may be relatively low for a simple marker-only construct such as OX1138B but will nonetheless make long-term persistence of the marker in the field highly unlikely.

Under extreme conditions, with temperatures regularly exceeding 40°C, DsRed2 provided a reliable and easily screened marker, and the molecular marker was extremely durable and identified moth type correctly in all analyses. These traits would be a valuable asset for the existing pink bollworm SIT programme particularly as the program approaches eradication of the pest. These results also demonstrate the potential value of DsRed2 and other fluorescent proteins as markers for SIT in general.

## Materials and Methods

Moths of the two strains – OX1138B and wild-type (APHIS) – were reared and sterilised using methods similar to those employed in the SIT programme. All moths used were reared on standard PBW diet [29] containing the Solvent Red 26 dye. OX1138B moths were reared in a quarantine laboratory in Phoenix, Arizona (Center for Plant Health Science and Technology, CPHST). APHIS moths were reared in the adjacent SIT mass-rearing facility. Pupae were sexed, and males were allowed to eclose. They were then irradiated with 200 Gy [13]. To simulate field conditions and to avoid adding additional treatment effects (such as freezing to kill the moths), live male moths - which in the field would be the recaptured sex on these pheromone-baited traps - were placed individually in grids of a marked trap using forceps (Figure 2).

For each trial Period, four traps were set up as follows: each trap received a total of 60 moths, of which 3–7 were APHIS moths and the remainder OX1138B moths. We used these proportions of the two moth types to approximately reflect that of typical trap recaptures in the pink bollworm SIT programme, in which released moths typically outnumber wild moths. The relative position of moths from the two strains was unique for each trap. The position of each moth was recorded and each trap was marked with a unique number on the outside. The moths were screened under a fluorescent microscope for the DsRed2 marker. In order to provide a blind test of the marker, trap preparation and moth screening were conducted by different personnel. Three of these traps were then placed on stakes within a row of cotton plants growing inside a screened quarantine cage (3 m×3 m×2.5 m), outdoors. The remaining trap was stored in the laboratory at 26°C. All of the traps were then periodically screened for DsRed2 fluorescence in the moths (Figure 1), and then replaced in the cage/laboratory.

Trap screening results were then passed to the scientist who set up the trap to compare them with the true identities of the moths. The process continued until results showed that correct identification of DsRed2-marked moths was below 50%. At later stages of each Period, when the marker sometimes became difficult to identify, some moths were broken up on the trap to view internal tissues.

This process was repeated four times, over four consecutive periods between 2^nd^ July and 19^th^ November 2010 (Period 1, 2^nd^–29^th^ July; Period 2, 30^th^ July to 27^th^ August; Period 3, 8^th^ September to 13^th^ October; Period 4, 15^th^ October to 19^th^ November). Temperature and humidity data, measured every 30 min (Hobo Pro, Onset Computer Corp.), were collected inside the cage for the duration of the experiment.

**Figure 6:**
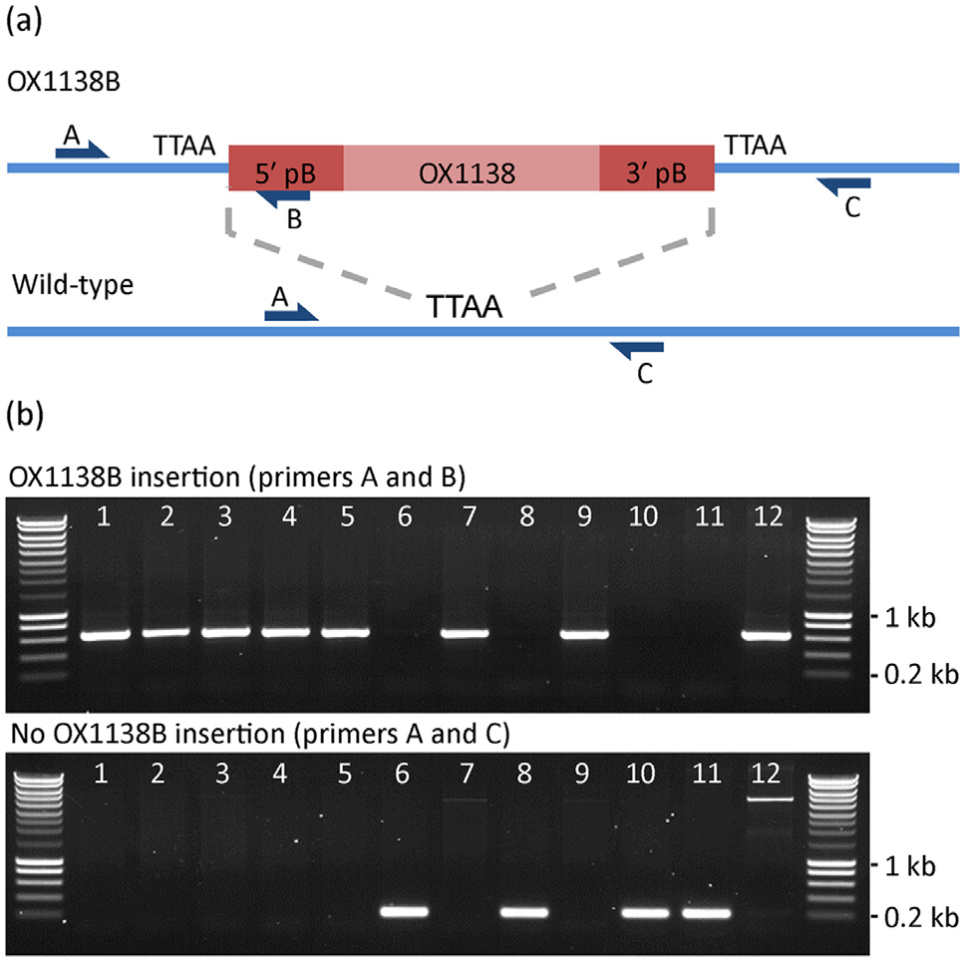
Genotyping moths by PCR. (a) Schematic diagram showing genotyping PCR reactions for the OX1138B transgene insertion and its wild-type counterpart. The junction of the OX1138B insertion site in the wild-type sequence is indicated by the TTAA nucleotide sequence (*piggyBac* transposase recognition sequence which is duplicated on insertion of the *piggy*Bac transposon). Primers and binding sites for primers A, B and C are indicated. PCR reactions containing primers A and B will amplify a fragment of 580 bp from OX1138B genomic DNA and no fragment from wild-type genomic DNA. PCR reactions containing primers A and C will amplify a fragment of 336 bp from wild-type genomic DNA and no fragment from OX1138B-homozygous genomic DNA. A moth that is heterozygous for the OX1138B insertion – carrying both the OX1138B and wild-type loci – would yield the amplified fragments in both PCR reactions. In OX1138B-positive genomic DNA, primers A and C would theoretically yield an amplified fragment, but the distance is usually too great for the PCR to amplify. (b) Gel images showing PCR genotyping results of moths from an experimental field cage trap (samples 1–10), a known wild-type moth (sample 11) and a known OX1138B-homozygous moth (sample 12). PCR for the transgene insertion yields the 580 bp fragment OX1138B moths (samples 1–5, 7 and 9) and PCR for the wild-type (no transgene insertion) locus yields the 336 bp fragment in APHIS moths (samples 6, 8 and 10). In the latter PCRs, amplification of the whole sequence spanning the transgene insertion is achieved, as seen in the reaction for sample 12.

At the completion of each replicate, the traps were stored at −20°C to preserve the moths’ genomic DNA. At the conclusion of the study, two traps from each period were sent, chilled, to Oxitec laboratories in the UK for PCR analysis. From each period, genomic DNA was extracted (GeneJET® genomic purification kit, Fermentas) from 75 moths (one whole trap plus 15 moths randomly selected from other traps using www.random.org), and each sample was genotyped by PCR for the presence of two sequences (Figure 6) [2 min at 94°C, 3× (10 s at 95°C, 1 min at 62°C, 2 min at 72°C), 27× (10 s at 95°C, 30 s at 62°C, 55 s at 72°C), and 5 min at 72°C] - one spanning the 5′ junction of the OX1138B insertion (primers 5′-CTGCTCGGGCGAGCGTATATAGAC-3′ and 5′-CTCTGGACGTCATCTTCACTTACGTG-3′) and the other spanning the wild-type genomic insertion site of the transgene (amplifies a fragment when no transgene is present; primers 5′-CTGCTCGGGCGAGCGTATATAGAC-3′ and 5′-CCGCCGTCATTTCTACATTAGTAAGA-3′) - which we used to identify OX1138B and APHIS moths, respectively. For the insertion-amplifying reaction, DNA extracted from an OX1138B moth would result in an amplified fragment of 580 bp. The absence of this fragment, together with amplification of the wild-type fragment (336 bp) indicated an APHIS or wild moth. PCR screening was also conducted on a double-blind basis: personnel conducting the molecular analysis had no prior knowledge of the identity (OX1138B or APHIS) of each moth.
